# Old Age Support in Urban China: The Role of Pension Schemes, Self-Support Ability and Intergenerational Assistance

**DOI:** 10.3390/ijerph16111918

**Published:** 2019-05-30

**Authors:** Lucille Aba Abruquah, Xiuxia Yin, Ya Ding

**Affiliations:** School of Management and Economics, University of Electronic Science and Technology of China, No. 2006 Xiyuan Ave, West Hi-Tech Zone, Chengdu 611731, China; 201614110107@std.uestc.edu.cn (L.A.A.); 201521110108@std.uestc.edu.cn (X.Y.)

**Keywords:** life satisfaction, intergenerational interaction, China’s urban elderly, pension reform, personal finance, public pension scheme, Government and Institution Pension, Enterprise Employee Basic Pension, Urban-Rural Social Pension Scheme

## Abstract

With the aim of probing into the life satisfaction of retired urban elderly in China with respect to old age support systems, this study examines the effect of pension reform with its existing inequalities across demographic and social groups on the life satisfaction of retired urban residents. The complementary role of intergenerational assistance and self-support on the life satisfaction of beneficiaries and non-beneficiaries of the pension scheme was analyzed using an ordered logit regression model with 2015 national representative data from China’s Health and Retirement Longitudinal Survey. Our sample consists of a cross-sectional data set of 3815 retired urban elderly aged 60 and above. The empirical results depict that though enjoying benefits from the public pension scheme generally enhances life satisfaction, beneficiaries of the Government and Institution Pension and Enterprise Employee Basic Pension are more advantaged than beneficiaries under the Urban-Rural Social Pension Scheme. The pension inequalities existing at provincial levels and across social groups such as gender and residence registration status also affect life satisfaction adversely. Women and rural ‘Hukou’ registered retired urban residents are at an apparent disadvantage. Getting financial and emotional support from children broadly improves life satisfaction. Non-beneficiaries of the public pension benefit more from the financial support of children than public pension beneficiaries. There is also a positive effect of cohabiting with children on life satisfaction when retired urban residents are single as compared to being married. Financial and physical self-support ability in forms of good health, home ownership and wealth management enhance life satisfaction significantly. However, largely, retired urban elderly have a higher life satisfaction when they are financially independent of children and are supported by state pension schemes. Our findings indicate that self-support ability of the elderly together with pension benefits are more effective in enhancing the life satisfaction of retired urban elderly in China. It is recommended that government institute policies to promote personal finance initiatives by the elderly while improving the pension scheme and reducing pension inequality.

## 1. Introduction

The aftermath of China’s one-child policy together with the striking rise in life expectancy and declining fertility rate has resulted in a disproportionate rapid rise in its aging population. Over the past three decades, China has undergone several pension reforms to curb the surging concerns about its aging population and economic imperatives [1]. In recent years, the pension scheme has become more accessible to a majority of its citizens as it has evolved from a state-employer model to a state-society one [2]. Though the current pension scheme has achieved wide coverage, it has brought about new inequalities as it is run at provincial instead of at national level. Even across the various provinces, the pension scheme is segregated across rural and urban areas, as it is also run based on the household registration system [2]. The household registration system, also known as the “Hukou” system, provides citizens with a legal document, categorizing them into rural Hukou and urban Hukou [3]. Rural Hukou status is designated to citizens originally residing in towns and villages, while urban Hukou status is assigned to citizens residing in urban cities. Getting a rural or urban Hukou status is largely dependent on the Hukou status of an individual’s parents. The Hukou status can however be changed in certain circumstances such as marriage and purchasing of a house. As a result of the stratification of the current pension system, residents of large cities as well as those who hold an urban Hukou status are at an advantage compared to residents of smaller cities and those who hold rural residence status [4]. Rapid rural-urban migration and provincial variations in the pension scheme have recently made inequalities across residence status and demographic location more apparent as many rural-urban migrants do not enjoy urban social welfare benefits [5,6].

Complementary to the public pension scheme are other forms of old age support, such as intergenerational support and self-support. Intergenerational assistance, which sits at the core of the Chinese cultural value of filial piety, has been instrumental in providing a safety net for the retired elderly amidst the pension reforms [7]. Intergenerational assistance can be categorized into financial and emotional support from children. Self-support through personal finance, homeownership and wealth management has also gained popularity among senior citizens giving retired elderly the capacity to invest towards their old age [8]. Additionally, being in a good health condition enhances the elderly’s ability to physically support themselves, hence reducing the burden on children. The integration of the public pension scheme together with intergenerational support and self-support is likely to contribute towards a secure old-age support for the retired elderly rippling down to their perception of wellbeing. However, the question of a trade-off between the current pension scheme and other forms of old-age support arises.

This study aims at analyzing the effect of three major types of public pension schemes and the existing inequalities at provincial levels and across demographic and social groups on the life satisfaction (LS) of retired elderly. The effect of non-governmental forms of support, i.e., intergenerational support and self-support and the resultant trade-off with the pension scheme on LS, is also analyzed across subgroups of public pension beneficiaries and non-beneficiaries. The effect of the aforementioned forms of old-age support on the LS of retired urban residents are empirically analyzed using an ordered logit regression model with 2015 national representative data from China’s Health and Retirement Longitudinal Survey. Based on our findings, this paper identifies the deficiencies in China’s current public pension scheme and further assesses the implications for future policy action.

## 2. Public Pension Scheme, Intergenerational Assistance and Self Support

Based on the objectives of this study, we first discuss the transitions in China’s public pension scheme as well as the emerging role of intergenerational assistance and self-support ability in old age security and life satisfaction, making reference to both international and domestic literature.

### 2.1. Pension Reform in China

The rapid increase in China’s aging population since 2000 has made providing social safety nets, especially a sustainable pension scheme, a rising concern for the government [9,10]. Thus, China’s pension scheme has undergone extensive reforms in recent years, starting from employee insurance to social insurance [11,12]. During the initial stages of the urban employee pension scheme, just a limited number of employees were beneficiaries of the scheme as benefits centered mainly on employers’ contributions regardless of being a state-owned enterprise or private enterprise [13]. Many industries that had gone bankrupt at the time could not give pension benefits to their retired employees, which resulted in protests and riots by retired employees, leading to social unrest and disruption [4]. This led to the re-evaluation of the pension scheme by the government to ensure stability. Subsequently, a partial funding plan was introduced. This was a combination of a two pillar scheme; namely, the pay-as-you-go (PAYGO) scheme and a specified subscription scheme, financed by both employers (20%) and employees (8%) of gross monthly earnings [4,14,15,16]. This partial funding pension plan was seen to be flexible, reaping the full benefits of both plans and at the same time lessening their demerits. This greatly increased the number of urban workers benefiting from the scheme. However, this scheme faced some drawbacks due to the digression of the accumulated funds by local governments to address the pressing obligation of assisting pensioners who were not contributors to the pension pool [4,14].

Distinct from the urban employees’ pension scheme, the rural pension insurance was established on one pillar: a specified subscription plan where rural dwellers would voluntarily decide to pay an amount of money, not exceeding RMB 20 monthly, quarterly or yearly. The rural pension scheme benefits were based on the accumulation of pension contributions and return on investments [4]. Compared to the urban pension scheme, the rural pension scheme faced a delimitation of a pooling of funds leading to inequality in pension benefits [4,17]. Starting as a pilot project, the rural pension insurance scheme failed, with about a third of its participants dropping out in 2004 due to the drawbacks that were not overcome [4,18].

A new pension scheme evolved after 2004, which was geared towards providing basic non-contributory pension insurance. It was based on the grounds of equity and equality among citizens, especially citizens not covered by the urban employees’ pension scheme and the rural social pension scheme [2]. The new basic pension scheme took off in 2008, after a successful pilot experiment in Baoji, a city in the Shaanxi province [4]. Since 2015, further policy changes, such as merging the rural and urban residents pension scheme, have been effected to make China’s pension scheme more equitably beneficial to its citizens [19]. China’s public pension insurance system is currently made up of three pillars namely Enterprise Employee Basic Pension (EEBP), the Urban-Rural Resident Social Pension (URRSP) and the Government and Institution Pension (GIP) [2]. The EEBP is a scheme established by firms for their employees, where both employers and employees make contribution towards the pension fund. URRSP consist of a basic residents’ pension scheme, which covers the unemployed urban dwellers and a large percentage of rural dwellers. The urban and the rural residence pensions are run as distinct schemes in most provinces. These two schemes are not financed by employers’ contributions but instead financed by the central and local government [2,4]. On the other hand, the GIP, also known as the civil servants’ pension scheme, was created for workers of government institutions. The characteristics of the GIP are similar to the EEBP but these two schemes have not yet been merged.

Despite certain drawbacks such as low benefit amount, provincial disparities, insufficient funding and limited pension investment choices, China’s pension schemes, to some extent, have provided a safety net to retirees, which is likely to improve the life satisfaction and quality of life during retirement [4,10]. While retirement itself has been reported to have a multifarious effect on life satisfaction across countries and time spans [20,21,22,23,24], pension insurance coverage on the other hand, has been found to be a likely determinant of the cross-country variations in the relationship between life satisfaction and retirement [24,25]. Generally, pension schemes tend to have a positive effect on the life satisfaction of the elderly [7,24,26,27,28]. A study on the moderating effect of income and endowment insurance policy values on retirement life support found that the provision of better quality pension schemes improves the life satisfaction of employees both during and prior to retirement [26]. Calvo found that the government has a major role to play on the dynamics between pension schemes and life satisfaction. His study concluded that a greater commitment to providing a better pension policy for its citizens improves life satisfaction significantly [27]. Hye-Won Kim also reported that public social safety nets such as pension schemes have a positive impact on the life satisfaction of Korean elderly [28]. Additionally, research on the impact of a reform in the Dutch public pension scheme revealed that the reform had an adverse effect in the form of increased depression on respondents who experienced lower pension benefits due to the reform [29]. Most research relating to the impact of pension schemes on life satisfaction focuses on public pension schemes without taking into consideration the inequalities existing across the types of pension scheme. Studies on the trade-off between public pension schemes and intergenerational support on life satisfaction are also limited. We therefore bridge this research gap by analyzing the effect of pension programs, in its entirety, on the life satisfaction of urban dwellers in China.

### 2.2. Intergenerational Assistance and Self Support Ability

The Confucian and Buddhist norm of filial piety, which is deeply engrained into Chinese culture, has over the years provided the basis for old age support. The conventional norm that old age support was a responsibility of the family, especially children, resulted in the conception of an individual raising children in anticipation of old age to be predominant in China. In the past, where formal old age support systems were limited and lacked wide coverage, many retired elderly depended on their children for financial and emotional support [30]. Support for retired elderly became a real burden on children during the era of the one-child policy. In recent times, though old age support from children still persists, it is undergoing dramatic changes due to reasons such as changes in the traditional family structure, migration, the prevalence of public social safety net policies and the rising trend of personal financing initiatives especially in the urban areas [31]. The study on the effect of intergenerational assistance from children on the life satisfaction of urban elderly in the midst of these current social changes poses a special importance. Generally, assistance from children positively affects the life satisfaction of the elderly [30,32,33,34,35]. Some studies find that though financial and emotional support enhances life satisfaction, emotional support plays a larger role in the perception of wellbeing among the elderly [36,37,38]. Other findings indicate that the positive effect of intergenerational assistance on perceived wellbeing of the elderly is stronger among the elderly who continue to adhere to more conventional and traditional norms of filial piety [38]. Research on intergenerational support in the form of living arrangements is however contradictory [38,39,40,41]. Despite the fact that many studies have investigated the impact of intergenerational interaction on the life satisfaction of the elderly, these studies are usually conducted independent of the rising social changes raised above.

For instance, there has been a rising trend of personal financing initiatives and savings by the elderly, such that most elderly can afford to be independent from their children during retirement. Data from the yearly household survey administered by the National Bureau of Statistics on the saving rates of households report a remarkable increase in the saving rates of Chinese citizens since the 1990s [42]. The major reasons suggested by literature for the current increase in household savings are the rising economic uncertainties and transitions in the social welfare system. This could be due to rising sensitization on the importance of savings as a source of financial security. Household personal savings if invested into ventures such as purchasing a house, buying of stocks and bonds and establishing self-employment ventures consequently earn returns, which contribute to future economic security and wealth [43,44]. Both domestic and foreign studies have found a positive effect of asset ownership and personal financing on life satisfaction [45,46,47,48,49]. With research relating to China, findings from a panel data analysis of the China Household Financial Survey indicated that growth in the personal assets of an individual could improve happiness, whereas increase in debt is detrimental to an individual’s perception of happiness [50]. Similarly, [41] found that owning housing property is also an important determinant of happiness in Urban China. Conversely, households with fewer personal assets tend to have lower levels of happiness as compared to their counterparts who have a wide accumulation of assets, even when income is controlled for [51]. International research regarding the link between asset ownership and life satisfaction also depict similar findings. A study on the life satisfaction of aboriginal populations in Malaysia found a positive relationship between an increase in wealth and life satisfaction [52]. Likewise, focusing on the effect of assets and debt on the life satisfaction of a nationally representative sample in Korea [53] indicated that changes in personal asset ownership influences life satisfaction significantly. A similar study on Singapore also concluded that real assets as well as monthly household income are essential predictors distinguishing moderate life satisfaction from high life satisfaction [54]. The financial ability of individuals to support themselves goes hand in hand with their physical ability to do so. Especially for the elderly, being in good health affords them the ability to be physically independent instead of relying on family to carry out their day-to-day activities. Being of good health and having the ability to carry out instrumental daily activities is widely found to have a significant effect on life satisfaction [20,55,56,57]. The effect of retired urban residents’ ability to physically support themselves measured using health status as a proxy on the life satisfaction of the retired elderly in China will also be assessed in this study.

## 3. Materials and Methods

### 3.1. Data and Variables

This study uses data from the China Health and Retirement Longitudinal Survey (CHARLS). With its baseline national wave beginning in 2011, CHARLS collects representative samples on demographics, family structure, health status and social welfare policies of Chinese individuals and households aged 45 and above, nationwide. Respondents are tracked every two years. The 2011 baseline survey consisted of about 10,000 households and 17,500 individuals in 28 provinces, 150 counties and 450 villages. Prior to the baseline survey in 2011, a pilot study which collected data from about 2685 individuals and 1570 households in 48 villages and 16 counties was carried out in the Zhejiang and Gansu province in 2008. The CHARLS survey employs a Multi-Stage Stratification with Probability Proportional to Size (PPS) sampling method [58,59]. The CHARLS survey has so far collected three waves of data since 2011. This study uses the Wave 3 of the CHARLS survey conducted in 2015 and made public in 2017. Our initial sample consists of a cross sectional data of 3815 retired urban residents aged 60 years and above. Retired urban residents in this context include retired local residents as well as rural-urban migrants. See Zhao et al. for further information on the CHARLS survey [58].

#### 3.1.1. Dependent Variable

**Figure 1:**
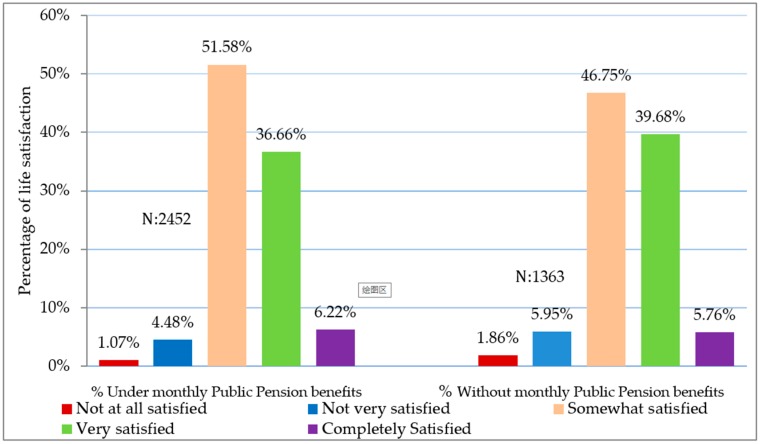
Distribution of life satisfaction among monthly beneficiaries of public pension and non-beneficiaries.

The dependent variable, self-reported LS, which serves as a good proxy for true LS, is based on the question in the CHARLS survey: “Please think about your life-as-a-whole. How satisfied are you with it?” The responses were rated on a five-point scale ranging from 1-completely satisfied to 5-completely dissatisfied. For consistency and easy comparison with the independent variables, the self-rated life satisfaction scale was reversed with 1 and 5 representing not at all satisfied and 5 completely satisfied, respectively. For retired urban elderly in 2015, about 6.05%, 37.74% and 49.50% of respondents reported completely satisfied, very satisfied and somewhat satisfied with their lives, respectively, while 5.05% and 1.66% of respondents reported not very satisfied and not at all satisfied. This distribution of urban life satisfaction is consistent with literature purporting that most individuals lie in the positive scale of somewhat satisfied to completely satisfied with their lives [60]. Figure 1 provides further insight into the percentage distribution of life satisfaction among respondents who are under the public pension system and those who are not. Analyzing the extremes of the distribution of life satisfaction, the percentage proportion of respondents in the “completely satisfied with life” category was higher by 0.46% for respondents under pension scheme coverage in comparison to respondents not under the pension program. Additionally, in the “not at all satisfied” bracket, the percentage proportion of pension beneficiaries was lower by 0.79% in contrast to non-beneficiaries. These findings are in support of our premise that public pension promotes life satisfaction of retired urban residents.

#### 3.1.2. Explanatory Variables

##### Public Pension and Pension Inequality

The pension scheme is measured by four variables. The dummy variable, “Public Pension” denotes whether or not the respondent receives regular monthly benefits from any of the pension schemes. The monthly benefits received from the three major types of pension schemes are denoted by the variables “Pension benefit from GIP”, “Pension benefit from EEBP” and “Pension benefit from URRSP” representing GIP, EEBP and URRSP, respectively. Though there is another type of non-contributory pension known as the Advanced Age Allowance (AAA), which targets elderly above 80 years, this pension has limited coverage in only 18 provinces; hence, it was excluded from our analysis [2]. Out of the sample of 3815 retired urban residents, about 7% received regular monthly benefits from the GIP, 20% were regular monthly beneficiaries of EEBP and 38% were beneficiaries of URRSP. The remaining respondents were either beneficiaries of the AAA or excluded from any of the pension schemes. The Pension inequality denoted by “Pension Income Inequality” is measured using the Gini coefficient of monthly pension income at provincial level. The monthly pension benefits of respondents in a particular province are used to calculate the Gini coefficient for the said province. There are 27 provinces, thus 27 unique Gini coefficients each representing a province. Pension inequality is further analyzed in terms of the degree of its effect on the LS of gender and residence registration status represented by the variables “Pension Income Inequality × Gender” and “Pension Income Inequality × Hukou Status”, respectively. “Pension Income Inequality × Gender” is derived from the interaction between the calculated Gini coefficient and gender. Likewise, “Pension Income Inequality × Hukou Status” is an interactive term generated from the Gini coefficients and residence status.

##### Intergenerational Assistance and Self Support Ability

Having children, cohabiting with children as well as receiving financial and emotional support from children are used as measures for intergenerational support. We first assess whether having a child has an effect on life satisfaction by introducing the dummy variable “Have at least one child”, where 1 represents the respondent having at least one child and 0 otherwise. “Cohabiting with children” is a dummy variable, where 1 represents whether the respondent lives in the same apartment with any of their children and 0 otherwise. The degree of effect cohabiting with children has on LS in terms of health status and marital status of retired urban residents is also analyzed using the interactive terms, “Health status based on ADL and IADL × Cohabiting with children”, “Health status based on chronic disease × Cohabiting with children” and “Cohabiting with children × Marital Status” representing the different measures of health status and marital status, respectively. The effect of frequent communication and financial assistance on life satisfaction is also measured with two variables: “Communication with children” and “Financial assistance from children”. Interactive terms “Pension benefits from GIP × financial assistance from children”, “Pension benefits from EEBP × financial assistance from children” and “Pension benefits from URRSP × financial assistance from children” are also introduced to measure the effect of a trade-off between receiving regular pension benefits from the three types of pension schemes and financial assistance from children on life satisfaction.

Retired urban elderly can support themselves through various ways, such as returns from savings and investment, income from self-employed activity, proceeds from renting out owned apartment, etc. The variable “Personal financing” represents proceeds received from all these personal financing channels. Additionally, living in ones owned apartment instead of renting provides a sense of security and an indication of ability to support oneself as far as accommodation is concerned. The dummy variable “Residence Ownership” was generated with 1 representing whether the respondent lives in apartment fully or partially owned by them and 0 otherwise. Being in good health condition also provides the elderly the ability to support themselves physically instead of relying on family in their daily activities. The CHARLS survey makes use of the respondent’s ability to perform general activities of daily living (ADL), instrumental activities of daily living (IADL) and whether the respondent suffers from any chronic disease as a measure of health. In this study, health status is divided into two separate variables i.e., a combination of ADL and IADL as one variable and whether the respondent suffers from any chronic disease as the second variable denoted by “Health status based on ADL and IADL” and “Health status based on chronic disease”, respectively. The questions on ADL and IADL constituted of 21 questions on activities of daily living and instrumental activities of daily living. Responses to these questions are measured on a four-point scale: 1 = ‘Cannot do it’, 2 = ‘Have difficulty and need help’, 3 = Have difficulty but can still do it’ and 4 = ‘Do not have any difficulty’. The variable “DL_Health” is a continuous variable measured using the weighted sum based on the number of questions the respondent answers in relation to ADL and IADL. The dummy variable “Health status based on chronic disease” also denotes whether the respondent suffers from any chronic disease, where ‘1’ represents whether respondent suffers from at least one of the specified chronic diseases and ‘0’ where respondent does not suffer from any of the specified chronic diseases. Further details, including definition of each variable used in this study, are illustrated in Table 1.

##### Control Variables

Considering that previous literature has identified a plethora of other determinants of life satisfaction such as age, gender, level of education and marital status to be crucial in LS research, we include these determinants as control variables in this study. The variable “Age” represents the age of the respondent ranging from 60 years to 105 years. A dummy variable “Gender” is also introduced, with 1 denoting male and 0 female. The variable “Education” measures the level of education of the respondent, on a scale of 1 to 10 with 1 representing illiterate and 10, Master’s degree. “Education2” represents the square of the variable “Education”. “Marital status” is a dummy variable representing the marital status of the respondent with 1 denoting married and 0 unmarried. The variable “Hukou status” denotes whether or not the respondent holds an urban residence status.

### 3.2. Analysis

In life satisfaction research, an ordered logit or probit regression model is an appropriate measure due to the categorical nature of the dependent variable. The ordered logit regression also takes into consideration the “floor and ceiling effects” peculiar to categorical variables, which may not be accounted for in other regression models [61]. This study uses an ordered logit regression model analogous to the one below:

(1) $$\begin{array}{cc} LS_{i}^{*}=\alpha +\;\beta & {\left(Pub\_Pen\right)}_{i}+\beta {\left(Gini\_Pen\right)}_{i}+\;\beta {\left(Int_{Sup}\right)}_{i}+\beta \;{\left(Self\_Sup\right)}_{i}\\ & +\beta \;{\left(Pers\_X\prime tics\right)}_{i}+\;\varepsilon_{i\;}\;\left(i=1,\ldots I;\;\beta =\left(\beta_{1},\;\beta_{2},\ldots ,\beta_{k}\right)\right) \end{array}$$

(2) $$LS_{reported}=Κ↔\lambda_{\kappa }\leq LS_{i}^{*}\leq \lambda_{\kappa +1}$$

In the above model, *LS_reported_* is the self-reported level of life satisfaction of an individual *i*, which serves as a good proxy of LS_i_*, the true-life satisfaction, which is an unobserved latent variable as shown in Equation (2). β is a set of coefficients representing a row vector whose number of elements depends on the number of variables in each set. *Pub_Pen_i_* represents the set of public pension related explanatory variables. Similarly, *Gini_Pen_i_, Int_Sup_i_* and *Self_Sup_i_* represent sets of explanatory variables related to pension inequality, intergenerational support and self-support ability, respectively. *Pers_X’tics* represent personal characteristics of the respondent such as age, gender, level of education, residence status and marital status, which also serve as control variables of the model. $\lambda_{\kappa }$ in Equation (2) is the optimal cut-point. $\varepsilon_{i}$, is an error term that has a logit distribution.

We ran a stepwise ordered logit regression model, where we first analyzed the full sample with the basic model in Equation (1). We subsequently introduced the interactive terms *Gini_Gender_i_* and *Gini_Hukou_i_* to analyze the effect of the pension inequality on the life satisfaction of gender groups and ‘Hukou’ (resident registration) status, respectively. This is shown as Equations (A1) and (A2) in the Appendix A.

Finally, we divided the full sample into subgroups of public pension beneficiaries and non-beneficiaries and analyzed the varied effect of intergenerational assistance and self-support ability between the two subgroups. Equations (A3) and (A4) in Appendix A represent the subsamples of non-beneficiaries and beneficiaries of the pension scheme, respectively. We analyzed the trade-off between regular pension income from the three public pension schemes (GIP, EEBP and URRSP) and financial assistance from children and their effect on life satisfaction by including the interactive terms “*ChildTransfer_GIP_i_*”, “*ChildTransfer_EEBP_i_*”, and “*ChildTransfer_URRSP_i_*”. Additionally, in both Equations (A3) and (A4), the interactive terms “*ChildCohab_DLHealth_i_”, “ChildCohab_ChrDisease_i_*” and “*ChildCohab_Marital_i_*” were introduced to measure the degree of effect cohabiting with children has on LS in terms of the two proxies of health status and marital status of retired urban residents, respectively.

Preceding the regression analysis, the variance inflation factor (VIF) was used to test for multicollinearity. The VIF did not detect the existence of multicollinearity as seen in Table A1 in Appendix B. All Interactive terms were centered to correct the high correlation, which is an inevitable problem when dealing with interactive terms. We also applied robust standard errors to relax the assumption of homoscedasticity of the ordered response model.

## 4. Results and Discussion

### 4.1. Ordered Logit Analysis

An ordered logit regression is used to analyze the impact of old age support on life satisfaction. Table 2 illustrates the ordered logit analysis for the effect of public pension scheme, pension inequality, intergenerational interaction and self-support ability on the life satisfaction of retired urban residents. The sample of retired urban residents were divided into two sub-samples, i.e., pension beneficiaries and non-beneficiaries, to further assess the varied effect of old age support systems on the life satisfaction among the two groups.

#### 4.1.1. Effect of Pension and Pension Inequality on Life Satisfaction of Retired Urban Elderly

Findings of the correlational effect of the public pension scheme on life satisfaction show that being on the public pension scheme generally enhances life satisfaction. However, when specific types of public pension schemes are considered, we found that whereas pension income from the GIP and the EEBP strongly promotes the life satisfaction of retired urban elderly, the URRSP does not show a statistically significant relationship with the life satisfaction of retired urban elderly. This is an indication of the inequality existing among the types of public pension schemes such that its positive effect on life satisfaction is not apparent.

The pension income inequality existing at the provincial level is negatively correlated with the life satisfaction of the retired urban residents. By introducing two interactive terms, to analyze the degree of correlational effect of pension inequality with respect to gender and Hukou status on life satisfaction, we found that women and rural Hukou registered retired urban residents are at an apparent disadvantage. Table 3 shows that the inequality existing in the pension scheme enhances the life satisfaction of retired urban residents who hold an urban Hukou by about 90 percentage points higher than rural Hukou holders. The existing pension inequality as a result of residence status is likely due to inadequate nation-wide adjustment systems, which has varied the pension benefits between provinces and counties, as well as between pension types. Similarly, the degree of effect of pension inequality with respect to gender shows that the pension inequality has a statistically significant positive effect on males by about 25 percentage points higher than female retired urban elderly. Though the public pension coverage is equal for both men and women, the benefit levels are lower for women as the system is run based on work, tenure and earning related structure. Due to the persisting problem of gender inequality in China’s workforce, women tend to earn lesser than men under the same job titles and working condition, and hence do not benefit as much as men from the pension scheme when they retire. Additionally, inadequate compensation is rendered to women who enter the job market late or take time off the labor market due to motherhood and other child-care activities. Some women also take up the duty of being house wives instead of entering the job market, and hence, only enjoy the URRSP, which has lesser pension benefits as compared to the other pension schemes.

#### 4.2.2. Effect of Intergenerational Support and Self Support on the Life Satisfaction of Retired Urban Elderly

With respect to intergenerational support, having at least one child has a statistically significant positive effect on the life satisfaction of retired urban elderly in China. This supports existing research where having a child promotes life satisfaction [62]. On the hand, cohabiting with children during retirement is detrimental to life satisfaction, though this correlational effect is not statistically significant. Frequent communication with non-cohabiting children greatly enhances the life satisfaction of these retired urban elderly. However, the relationship between financial assistance and the life satisfaction of retired urban elderly is generally not statistically significant. One reason could be that most retired urban elderly would like to be independent of their children when it comes to financial assistance, as they are able to support themselves and benefit from the public pension scheme. This finding is very different from rural China, where financial support from non-cohabiting children enhances life satisfaction [62]. This finding could be an indication that self-support ability through personal finance and wealth management has become prevalent in urban China as a result of retired urban elderly not wanting to be financially dependent on their children. This leads us to our findings on the effect of self-support ability on the life satisfaction of retired urban elderly.

The ability of retired urban elderly to support themselves both financially and physically enhances life satisfaction significantly among retired urban residents. Personal financing through earnings from self-employment, proceed from renting out owned apartment, household savings and investments has a positive effect on the life satisfaction of the retired urban elderly at a less than 10% level of significance. Ownership of current living abode also has a statistically significant positive effect on the life satisfaction of retired urban elderly by 18 percentage points higher compared to living in a rented apartment or cohabiting with children. This confirms to the premise that retired urban elderly prefer to be financially independent and enjoy the security that comes with living in ones owned apartment instead of having to pay rent or living with children. Additionally, physical support ability in the form of good health and capacity to carry out instrumental activities of daily living is positively correlated with life satisfaction at less than 1% level of statistical significance. On the other hand, suffering from a chronic disease is negatively correlated with the life satisfaction of retired urban elderly. The inability of a retired urban elderly to physically support themself may mean they rely on family especially children for help in carrying out day-to-day activities. Additionally, poor health may lead to high medical expenses, which may ripple down to spending pension benefits and personal wealth on health care, and consequently, financially and physically on relying children for support.

#### 4.2.3. Effect of Old Age Support on Life Satisfaction among Pension Beneficiaries and Non-Beneficiaries

Comparing the life satisfaction of pension beneficiaries and non-beneficiaries, Table 3 shows that both beneficiaries and non-beneficiaries are affected adversely by pension inequality. Similar to the full sample, pension benefits from GIP and EEBP have a significant positive relationship on the life satisfaction of pension beneficiaries, while URRSP does not have such an apparent effect on LS. Having a child and frequently communicating with children enhances the life satisfaction of both pension beneficiaries and non-beneficiaries; however, financial assistance from children has a significant positive effect on only non-beneficiaries. This is likely because non-beneficiaries of the pension scheme financially rely more on their children for support during retirement hence report higher life satisfaction when such support is frequent. Since pension beneficiaries receive regular monthly pension benefits, they do not have to rely on their children’s financial support for their livelihood. The interactive effect of receiving regular monthly pension benefits and financial assistance from children on LS was not statistically significant across all types of pension schemes. Similarly, the interactive effect between cohabiting with children and health status was also not statistically significant among pension beneficiaries and non-beneficiaries. On the other hand, the interactive effect between cohabiting with children and marital status on life satisfaction is positive and statistically significant. This indicates cohabiting with children has a strong positive effect on the life satisfaction of retired urban elderly when they are single compared to those who are married. In both sub-samples, the ability to support one’s self physically i.e., being in good health and financially through personal financing and owning current living abode enhances the life satisfaction significantly. The above comparison indicate that retired urban residents prefer to be both financially and physically independent from children through the help of the state or personal financing and wealth management initiatives instead of depend on their children. It is only when these retired urban residents are not fortunate enough to benefit from the public pension scheme that financial assistance enhances their life satisfaction. This is clear evidence of the importance of state support in the lives of retired urban residents and the need for the government to encourage personal financing initiatives among its citizens to safeguard their retirement and ease the burden on children in supporting their retired parent financially.

## 5. Conclusions

This study, to the best of our knowledge, is the first of its kind connecting the life satisfaction of retired urban elderly with pension inequality in China. It also contributes to research investigation on creating a social safety net for the increasing elderly population in China. We focused on the association of receiving regularly monthly benefits and the inequality existing across the three main types of public pension with life satisfaction. The varied effect of such inequality was analyzed across social subgroups. Additionally, the role of intergenerational support and self-support ability in the presence and absence of the public pension scheme on life satisfaction was also evaluated.

The empirical results for the full sample of retired urban elderly show that regular monthly income from all the public pension schemes increases life satisfaction. However, further analysis of retired elderly who are under the public pension scheme demonstrate that regular monthly income from the Government and Institution Pension and Enterprise Employee Basic Pension increases life satisfaction whereas the Urban-Rural Social Pension scheme does not have such apparent effect on life satisfaction. Additionally, the inequality under these three pension schemes is detrimental to life satisfaction in both the full sample of retired urban elderly and subsamples of public pension beneficiaries and non-beneficiaries. It can also be inferred from our findings that males and urban residents who hold an urban Hukou benefit more from the current public pension scheme in China than females and urban residents who hold a rural Hukou. Complementary to pension benefits, intergenerational support in the form of financial support and frequent communication with children enhances life satisfaction in both the full sample and among pension beneficiaries and non-beneficiaries. However, for the retired urban elderly under the pension scheme, emotional support from children through frequent communication is more beneficial to life satisfaction than financial assistance. Additionally, living in one’s owned apartment increases life satisfaction as compared to living in a rented apartment or cohabiting with children. Contrary to our hypothesis, there is no statistically significant trade-off between being a beneficiary of any of the types of pension schemes and financial support from children. The interactive effect between cohabiting with children and health status was also not statistically significant among both pension beneficiaries and non-beneficiaries. Our results, however, indicate a strong positive relationship between of cohabiting with children and life satisfaction when retired urban elderly are single compared to being married.

Unlike intergenerational assistance, self-support ability measured by good health, personal financing, wealth management and home ownership enhances the life satisfaction of retired urban residents in both the full samples and sub samples. Largely, retired urban elderly have a higher life satisfaction when they are financially independent from children and are supported by state pension schemes. Therefore, old age support should be built on an equal pension system and a balanced intergenerational support, as well as a good health and strong financial independence of the elderly.

The varied effect of these variables on life satisfaction is evident of the importance of the government to institute policies to meet the diverse needs of the aging population. We suggest that the different types of pension schemes should be integrated and structured on a state level instead of provincial level as well as placing priority on the retired less advantaged groups such as women, rural Hukou holders and elderly residents of small cities and counties to bolster equality. It is also imperative for government to institute policies to promote personal finance and wealth management initiatives as well as sensitize its citizens on the need for investments, which will spill over to provide a secure financial support for them when they retire. Policies that support subsidized-price housing and housing cash reliefs commonly known as “Jingji shiyong fang” and “Zhufang xianjin butie” respectively [11] should be enforced and made easily accessible under certain conditions to promote home ownership among retired elderly citizens. Additionally, the house property tax [11], which is currently in its pilot stage, if enforced nationwide, should be subsidized for the retired elderly who own houses to reduce the burden on having to pay large sums of taxes during their retirement.

Despite the pertinent findings made in this study there are however some identified limitations. Firstly, since a cross-sectional study was used in analyzing the effect of old age support on the life satisfaction of retired urban elderly, findings are susceptible to certain biases such that only correlational implications can be made between selected variables and life satisfaction. Therefore, we raise the caveat that findings should only be interpreted as correlational not causal. Secondly, though this study has demonstrated the various dynamics of old age support on the life satisfaction of retired urban elderly in China, there is still the need for future research to probe into other concerns regarding pension inequality, intergenerational support and self-support ability. We therefore suggest further research from the perspective of comparing pension inequality and its effect on the life satisfaction of retirees between urban and rural areas, across provinces with different economic development levels. In addition, potential research path could also be undertaken to explore the relationship between income, pension inequality and social status on life satisfaction with a full-age range sample since our research only focuses on retired urban residents aged 60 years and above.

## Figures and Tables

**Table 1:** Description and summary statistics of variables.

Variables	Definitions	Mean	Standard Deviation (SD)	Min	Max
Age	Age of the respondents	68.72	7.02	60	105
Education^2^	The square of educational level	16.47	15.23	1	100
Personal Financing	Individual assets including past year wages, income from self-employed activities of past year, proceeds from renting out owned apartment, savings of this household (deposit, cash) and financial capitals (market value of stocks and mutual funds, value of government bonds, value of public housing funds) measured in 1000 RMB	22.70	6.83	0	576
Health status based on ADL and IADL	Health status measured by the weighted sum of ADL and IADL ranging from 1 to 4	3.10	0.49	1.05	3.81
Communication with children	Frequency of communication with non-cohabiting children within a year.	20	12.80	0	56
Financial assistance from children	Financial support from non-cohabiting children with in a year (1000 RMB)	3.40	6.98	0	40
Pension Income Inequality	The Gini coefficient of monthly pension income on provincial level	0.73	0.11	0.18	0.89
Pension benefit from GIP	Amount of monthly benefits respondent receives from the Government and Institution Pension scheme (1000 RMB).	3.71	3.25	0.45	51.2
Pension benefit from EEBP	Amount of monthly benefits respondent receives from the Enterprise Employee Basic Pension scheme (1000 RMB).	2.43	0.91	0.14	8
Pension benefit from URRSP	Amount of monthly benefits respondent receives from the Urban-Rural Social Residents Pension scheme (1000 RMB).	0.32	0.54	0.058	4
		**N (%)**		**Min**	**Max**
Life satisfaction	1 = “not at all satisfied”; 2 = “not very satisfied”; 3 = “somewhat satisfied”; 4 = “very satisfied”; 5 = “completely satisfied”	1 (1.66%) 2 (5.05%) 3 (49.50%) 4 (37.74%) 5 (6.05%)		1	5
Education (Edu)	The highest level of education the respondent received; 1 = “illiterate”, 10 = “Master’s degree”	1 (19.45%) 2 (14.50%) 3 (21.68%) 4 (18.22%) 5 (14.76%) 6 (4.51%) 7 (3.80%) 8 (1.86%) 9 (1.21%) 10 (0.03%)		1	10
Gender	Gender of respondent, 1 = male, 0 = female	0 (51%) 1 (49%)		0	1
Marital Status	Marital status of respondent; 1 = “single”, 0 = “with alive partner”	0 (77.30%) 1 (22.70%)		0	1
Hukou Status	Residence status of respondent, where 1 denotes having urban residence status and 0 otherwise.	0 (45.16%) 1 (54.84%)		0	1
Residence Ownership	Ownership of current living house, with 1 representing ‘the current living house is entirely or partly owned by the respondent’ and 0 ‘otherwise’	0 (39.79%) 1 (60.21%)		0	1
Health status based on Chronic disease	Whether the respondent suffers from any chronic disease (physical, mental and cognitive), with 1 ‘denoting suffers from a chronic disease’ and 0 ‘otherwise’	0 (33.11%) 1 (66.89%)		0	1
Have at least one child	Whether the respondent has a at least one child, where 1 measures the respondent has children and 0 otherwise	0 (5.06%) 1 (94.94%)		0	1
Cohabiting with children	Whether the respondent lives in the same house with children or not, where 1 represents the respondent shares the same roof with their children and 0, otherwise.	0 (77%) 1 (23%)		0	1
Public Pension	Whether or not the respondent receives regular monthly benefits from any of the pension schemes.	0 (35.3%) 1 (64.27%)		0	1

Notes: ADL: Activities of Daily Living; IADL: Instrumental Activities of Daily Living; GIP: Government and Institution Pension; EEBP: Enterprise Employee Basic Pension; URRSP: Urban-Rural Social Residents Pension.

**Table 2:** Ordered logit estimation results of the effect of old-age support on life satisfaction.

Variables	Model 1	Model 2	Model 3
**Pension Inequality**			
Pension Income Inequality	−0.8576 *** (0.3262)	−1.0176 *** (0.3275)	−1.5752 *** (0.3542)
Pension Income Inequality × Gender		1.1582 * (0.6551)	1.3759 ** (0.6774)
Pension Income Inequality × Hukou status			1.3493 ** (0.7982)
**Pension**			
Public Pension	0.4123 *** (0.0963)	0.4339 *** (0.0953)	0.3374 *** (0.0946)
Pension benefit from GIP	0.2163 *** (0.0298)	0.2180 *** (0.0300)	0.2571 *** (0.0290)
Pension benefit from EEBP	0.2177 *** (0.0389)	0.2157 *** (0.0394)	0.2916 *** (0.0387)
Pension benefit from URRSP	0.0436 (0.0982)	0.0266 (0.0994)	0.0310 (0.0990)
**Self_Support_Ability**			
Personal Finance	0.0012 *** (0.0004)	0.0011 ** (0.0004)	0.0012 ** (0.0004)
Residence Ownership	0.4238 *** (0.0758)	0.4387 *** (0.0757)	0.4266 ** (0.0759)
Health status based on ADL and IADL	0.4505 *** (0.1501)	0.4249 ** (0.1489)	0.4497 *** (0.1463)
Health status based on chronic disease	−0.2989 *** (0.0669)	−0.2952 *** (0.0673)	−0.3109 *** (0.0675)
**Intergenerational_Support**			
Have at least one child	0.3910 ** (0.2125)	0.3856 ** (0.2122)	0.5368 ** (0.2145)
Cohabiting with children	−0.0828 (0.0865)	−0.0915 (0.0867)	−0.1156 (0.0873)
Financial assistance from children	0.0008 (0.0051)	0.0009 (0.0051)	0.0006 (0.0051)
Communication with children	0.0130 ***(0.0028)	0.0117 *** (0.0028)	0.0101 *** (0.0028)
**Control Variable**			
Age	YES	YES	YES
Education Education^2^	YES YES	YES YES	YES YES
Marital status	YES	YES	YES
Gender	YES	YES	YES
Hukou status	YES	YES	YES
**Regression Index**			
Wald Chi2	501.40	512.46	459.60
Prob > Chi2	0.00	0.00	0.00
Pseudo R2	0.06	0.06	0.05
Observations	3815	3815	3815

Robust standard errors in parentheses *** *p* < 0.01, ** *p* < 0.05, * *p* < 0.1.

**Table 3:** Estimation results for Pension scheme beneficiaries and non-beneficiaries.

Variables	Pension Beneficiaries	Non-Beneficiaries
**Pension Inequality and Pension Types**		
Pension income Inequality	−0.8856 ** (0.4012)	−1.0397 * (0.6474)
Pension benefit from GIP	0.1902 *** (0.0464)	
Pension benefit from EEBP	0.0959 *** (0.0539)	
Pension benefit from URRSP	0.0198 (0.1167)	
Pension benefits from GIP × financial assistance from children	0.0066 (0.0069)	
Pension benefits from EEBP × financial assistance from children	0.0086 (0.0053)	
Pension benefits from URRSP × financial assistance from children	0.0024 (0.0245)	
**Self_Support Ability**		
Personal Finance	0.0015 *** (0.0006)	0.0021 ** (0.0010)
Residence ownership	0.4115 *** (0.0940)	0.5483 *** (0.1446)
Health status based on ADL and IADL	0.6539 *** (0.2649)	0.3486 ** (0.1714)
Health status based on chronic disease	−0.2583 ** (0.0884)	−0.4493 *** (0.1427)
Health status based on ADL_IADL × Cohabiting with children	−0.7450 (0.6264)	−0.6879 (0.3444)
Health status based on chronic disease × Cohabiting with children	−0.2769 (0.0918)	−0.0126 (0.2921)
**Intergenerational_Support**		
Have at least one child	0.4117 *** (0.2632)	0.4205 ** (0.2398)
Cohabiting with children	−0.3353 ** (0.6953)	−0.1976 (0.3629)
Financial assistance from children	0.0052 (0.0093)	0.0305 *** (0.0101)
Communication with children	0.0078 ** (0.0035)	0.0149 *** (0.0048)
Cohabiting with children × Marital Status	0.4639 *** (0.2231)	0.7304 *** (0.2969)
**Control Variables**	YES	YES
Wald Chi2	404.15	219.07
Prob > Chi2	0.00	0.00
Pseudo R2	0.08	0.06
Observations	2452	1363

Robust standard errors in parentheses, *** *p* < 0.01, ** *p* < 0.05, * *p* < 0.1.

## Appendix A

(A1) $$\begin{array}{ll} LS_{i}^{*}=\alpha +\;\beta & {\left(Pub\_Pen\right)}_{i}+\beta {\left(Gini\_Pen\right)}_{i}+\;\beta {\left(Gini\_Gender\right)}_{i}+\;\beta {\left(Int\_Sup\right)}_{i}\\ & +\beta \;{\left(Self\_Sup\right)}_{i}\;+\;\beta \;{\left(Pers\_X\prime tics\right)}_{i}+\;\varepsilon_{i\;}\;(i=1,\ldots I;\beta \\ & =\left(\beta_{1}\;,\beta_{2,},\ldots ,\beta_{k}\right) \end{array}$$

(A2) $$\begin{array}{ll} LS_{i}^{*}=\alpha +\;\beta & {\left(Pub\_Pen\right)}_{i}+\beta {\left(Gini\_Pen\right)}_{i}+\;\beta {\left(Gini\_Gender\right)}_{i}+\beta {\left(Gini\_Hukou\right)}_{i}\\ & +\;\beta {\left(Int\_Sup\right)}_{i}+\beta \;{\left(Self\_Sup\right)}_{i}+\beta \;{\left(Pers\_X\prime tics\right)}_{i}+\;\varepsilon_{i\;}\;(i\\ & =1,\ldots I;\beta =\left(\beta_{1}\;,\beta_{2,},\ldots ,\beta_{k}\right) \end{array}$$

(A3) $$\begin{array}{ll} LS_{i}^{*}=\alpha +\;\beta & {\left(Gini\_Pen\right)}_{i}+\beta {\left(Self\_Sup\right)}_{i}+\;\beta {\left(ChildCohab\_DLHealth\right)}_{i}\\ & +\beta {\left(ChildCohab\_ChrDisease\right)}_{i}+\;\beta {\left(ChildCohab\_Marital\right)}_{i}\\ & +\beta \;{\left(Pers\_X\prime tics\right)}_{i}+\;\varepsilon_{i\;}\;(i=1,\ldots I;\beta =\left(\beta_{1}\;,\beta_{2,},\ldots ,\beta_{k}\right)\; \end{array}$$

(A4) $$\begin{array}{ll} LS_{i}^{*}=\alpha +\beta & {\left(Pub\_Pen\right)}_{i}+\;\beta {\left(Gini\_Pen\right)}_{i}+\;\beta {\left(ChildTransfer\_GIP\right)}_{i}\\ & +\;\beta {\left(ChildTransfer\_EEBP\right)}_{i}+\;\beta {\left(ChildTransfer\_URRSP\right)}_{i}\\ & +\beta {\left(Int\_Sup\right)}_{i}+\beta {\left(Self\_Sup\right)}_{i}+\;\beta {\left(ChildCohab\_DLHealth\right)}_{i}\\ & +\beta {\left(ChildCohab\_ChrDisease\right)}_{i}+\;\beta {\left(ChildCohab\_Marital\right)}_{i}\\ & +\beta \;{\left(Pers\_X\prime tics\right)}_{i}+\;\varepsilon_{i\;}\;(i=1,\ldots I;\beta =\left(\beta_{1}\;,\beta_{2,},\ldots ,\beta_{k}\right) \end{array}$$

## Appendix B

**Table A1:** Variance inflation factor (VIF).

Variables	VIF	1/VIF
Education	1.44	0.69
Age	1.43	0.70
Hukou Status	1.42	0.70
Pension benefit from EEBP	1.42	0.70
Communication with children	1.38	0.72
Marital status	1.38	0.73
Residence Ownership	1.38	0.73
Pension benefit from GIP	1.29	0.77
Cohabiting with children	1.29	0.78
Gender	1.26	0.79
Public Pension	1.24	0.81
Financial assistance from children	1.16	0.86
Personal Finance	1.16	0.86
Education^2^	1.14	0.88
Have at least one child	1.13	0.88
Pension Income Inequality	1.12	0.90
Pension benefit from URRSP	1.09	0.92
Health status based on ADL and IADL	1.09	0.92
Pension Income Inequality × Hukou Status	1.04	0.96
Health status based on chronic disease	1.04	0.96
Pension Inequality × Gender	1.06	0.97
Mean VIF	1.23	

## References

[1] Queisser M., Reilly A., Hu Y. (2016). China’s pension system and reform: An OECD perspective. Econ. Polit. Stud..

[2] Zhu H., Walker A. (2018). Pension system reform in China: Who gets what pensions?. Soc. Policy Adm..

[3] Liu Z. (2005). Institution and inequality: The hukou system in China. J. Comp. Econ..

[4] Liu T., Sun L. (2016). Pension Reform in China. J. Aging Soc. Policy.

[5] Kuang L., Liu L. (2012). Discrimination against Rural-to-Urban Migrants: The Role of the Hukou System in China. PLoS ONE.

[6] Huang Y., Guo F. (2017). Welfare Programme Participation and the Wellbeing of Non-local Rural Migrants in Metropolitan China: A Social Exclusion Perspective. Soc. Indic. Res..

[7] Ng S.T., Tey N.P., Asadullah M.N. (2017). What matters for life satisfaction among the oldest-old? Evidence from China. PLoS ONE.

[8] Van Dullemen C.E., Nagel I., de Bruijn J.M. (2017). Are the Chinese Saving for Old Age?. J. Popul. Ageing.

[9] Dong K., Wang G. (2016). China’s pension system: Achievement, challenges and future developments. Econ. Polit. Stud..

[10] Pozen R.C. (2013). Tackling the Chinese Pension System.

[11] Zhao L., Zhao L.T. (2013). Issues and options for social security reform in China. China’s Soial Development and Policy: Into the Next Stage?.

[12] Ringen S., Ngok K. (2013). What Kind of Welfare State is Emerging in China?.

[13] Feldstien M. (1999). Social Security Pension Reform in China. China Econ. Rev..

[14] Zhao Y., Xu J. (2002). China’s Urban Pension System: Reform and Problems. Cato.

[15] Wang Y. (2016). Understanding the Implementation Gap of China’s Urban Pension Scheme at the Level of Rural-Urban Migrant Workers.

[16] Xiqing G., Xiqing G. (2015). China’s Pension System: Its History and Future. Reinventing Retirement Asia: Enhancing the Opportunity of Aging.

[17] Vilela A. Pension Coverage in China and the Expansion of the New Rural Social Pension. https://www.refworld.org/pdfid/5301df5d4.pdf.

[18] Ministry of Finance The People’s Republic of China, Ministry of Finance Japan (2018). Joint Research Report on the Chinese and Japanese Pension Systems.

[19] Organization for Economic Co-operation and Development (2016). Pension at A Glance 2017: Country Profile-China.

[20] Gorry A., Gorry D., Slavov S. (2018). Does retirement improve health and life satisfaction?. Health Econ..

[21] Coe N.B., Zamarro G. (2011). Retirement effects on health in Europe. J. Health Econ..

[22] Smith J.P. (2013). Work, Retirement and Depression. J. Popul. Ageing.

[23] Charles K.K. (2002). Is Retirement Depressing? Labor Force Inactivity and Psychological Well-Being in Later Life.

[24] Fonseca R., Kapteyn A., Lee J., Zamarro G., Feeney K. (2013). A longitudinal Study of Well-being of Older Europeans: Does Retirement Matter?. J. Popul. Ageing.

[25] Kapteyn A., Lee J., Zamarro G. (2013). Does Retirement Induced Through Social Security Pension Eligibility Influence Subjective Well-Being? A Cross-Country Comparison. Mich. Retire. Res. Cent. Res. Pap..

[26] Li S., Zhang J., Shan L. (2018). How Life Satisfaction and Income Moderate the Effects of Endowment Insurance Policy Values on the Retirement Life Support Perceived by Employees in Private Firms. Acta Psychol. Sin..

[27] Calvo E. (2010). The impact of pension policy on older adults’ life satisfaction: An analysis of longitudinal multilevel data. Diss. Abstr. Int. Sect. A Humanit. Soc. Sci..

[28] Hye-Won Kim E. (2012). Public Support, Family Support, and Life Satisfaction of the Elderly: Evidence from A New Government Old-Age Pension in Korea.

[29] De Grip A., Lindeboom M., Monitizan R. (2011). Shattered dreams: The effect of changing the pension system late in the game. Econ. J..

[30] Cong Z., Silverstein M. (2008). Intergenerational support and depression among elders in rural China: Do daughters-in-law matter?. J. Marriage Fam..

[31] Guo M., Aranda M.P., Silverstein M. (2009). The impact of out-migration on the inter-generational support and psychological wellbeing of older adults in rural China. Ageing Soc..

[32] National Academy of Sciences Intergenerational Transfers (2001). Preparing for An Aging World.

[33] Chen J., Jordan L.P. (2016). Intergenerational support and life satisfaction of young-, old- and oldest-old adults in China. Aging Ment. Heal..

[34] Schwarz B., Albert I., Trommsdorff G., Zheng G., Shi S., Nelwan P.R. (2010). Intergenerational Support and Life Satisfaction: A Comparison of Chinese, Indonesian, and German Elderly Mothers. J. Cross. Cult. Psychol..

[35] Liang J., Zhang P., Zhu X., Qiao Y., Zhao L., He Q. (2014). Effect of intergenerational and intragenerational support on perceived health of older adults: A population-based analysis in rural China. Fam. Pract..

[36] Krause N., Ar H., Baker E. (1992). Providing support to others and well-being in later life. J. Gerontol. Psychol. Sci..

[37] Krause N. (1997). Anticipated Support, Received Support, and Economic Stress Among Older Adults. J. Gerontol. Geriatr. Res..

[38] Chen X., Silverstein M. (2000). Intergenerational Social Support and the Psychological Well-Being of Older Parents in China. Res. Aging.

[39] Brasher M.S. (2011). Living arrangements of older adults in China: The interplay among preferences, realities, and health. Res. Aging.

[40] Silverstein M., Cong Z., Li S. (2006). Intergenerational Transfers and Living Arrangements of Older People in Rural China: Consequences for Psychological Well-Being. J. Gerontol. Ser. B Psychol. Sci. Soc. Sci..

[41] Chyi H., Mao S. (2012). The Determinants of Happiness of China’s Elderly Population. J. Happiness Stud..

[42] Zhang Y., Shi L. (2011). East Asia Forum.

[43] McKernan S.-M., Sherraden M. (2008). Asset Building and Low-Icome Families.

[44] Caner A., Wolff E.N. (2004). Asset Poverty in the United States, 1984–1999: Evidence from the Panel Study of Income Dynamics. Rev. Income Wealth.

[45] Brown S., Gray D. (2014). Household Finances and Well-Being: An Empirical Analysis of Comparison Effects.

[46] Wolff E.N., Zacharias A. (2009). Household wealth and the measurement of economic well-being in the United States. J. Econ. Inequal..

[47] Cheng Z., Prakash K., Smyth R., Wang H. Housing Wealth and Happiness in Urban China. https://www.researchgate.net.

[48] Headey B., Muffels R., Wooden M. (2004). Money Doesn’t Buy Happiness.... Or Does It? A Reconsideration Based on the Combined Effects of Wealth, Income and Consumption.

[49] Landiyanto E.A., Ling J., Puspitasari M., Irianti S.E. Wealth and Happiness: Empirical Evidence from Indonesia. Proceedings of the 10th Indonesian Regional Science Association (IRSA) International Conference.

[50] Li J., Li H., Gan L. (2015). Household Assets, Debt and Happiness: An Explanation to “Happiness-Income” Puzzle. Nankai Econ. Stud..

[51] Deng S., Huang J., Sherraden M., Jin M. (2013). Asset opportunity for the poor: An asset-based policy agenda towards inclusive growth in China. China J. Soc. Work.

[52] Howell C.J., Howell R.T., Schwabe K.A., Howell J., Kurt A. (2014). Does Wealth Enhance Life Satisfaction for People Who Are Materially Deprived? Exploring the Association among the “Orang Asli” of Peninsular Malaysia. Soc. Indic. Res..

[53] Han C.-K. (2011). Song-lee Hon Assets and Life Satisfaction Patterns Among Korean Older Adults: Latent Class Analysis. Soc. Indic. Res..

[54] Hong S., Han C. (2014). Asset Impacts on Life Satisfaction in an Asset- Rich Country: Focusing on Older Adults in Singapore. Soc. Indic. Res..

[55] Puvill T., Lindenberg J., Craen A.J.M., De Slaets J.P.J., Westendorp R.G.J. (2016). Impact of physical and mental health on life satisfaction in old age: A population based observational study. BMC Geriatr..

[56] Sabatini F. (2014). The relationship between happiness and health: Evidence from Italy 1. Soc. Res. Med..

[57] Easterlin R.A. (2006). Life cycle happiness and its sources. J. Econ. Psychol..

[58] Zhao Y., Hu Y., Smith J.P., Strauss J., Yang G. (2014). Cohort profile: The China health and retirement longitudinal study (CHARLS). Int. J. Epidemiol..

[59] CHARLS China Health and Retirement Longitudinal Study. http://charls.pku.edu.cn/en/page/data/2015-charls-wave4.

[60] Frey S.B., Stutzer A. (2005). Happiness Research: State and Prospects. Rev. Soc. Econ..

[61] Agresti A. (2012). Analysis of Ordinal Categorical Data.

[62] Yin X., Abruquah L.A., Ding Y. (2019). Dynamics of Life Satisfaction Among Rural Elderly in China: The Role of Health Insurance Policies and Intergenerational Relationships. Sustainability.

